# Biochemical and Structural Insights into a Thiamine Diphosphate-Dependent α-Ketoglutarate Decarboxylase from Cyanobacterium *Microcystis aeruginosa* NIES-843

**DOI:** 10.3390/ijms241512198

**Published:** 2023-07-30

**Authors:** Zhi-Min Li, Ziwei Hu, Xiaoqin Wang, Suhang Chen, Weiyan Yu, Jianping Liu, Zhimin Li

**Affiliations:** 1 College of Chemistry and Materials, Jiangxi Agricultural University, Nanchang 330045, China; lzm_70@jxau.edu.cn; 2 College of Bioscience and Bioengineering, Jiangxi Engineering Laboratory for the Development and Utilization of Agricultural Microbial Resources, Jiangxi Agricultural University, Nanchang 330045, China; 3 Collaborative Innovation Center of Postharvest Key Technology and Quality Safety of Fruits and Vegetables in Jiangxi Province, Jiangxi Agricultural University, Nanchang 330045, China

**Keywords:** α-ketoglutarate decarboxylase, *Microcystis aeruginosa*, biochemical characterization, structural modeling

## Abstract

α-Ketoglutarate decarboxylase is a crucial enzyme in the tricarboxylic acid cycle of cyanobacteria, catalyzing the non-oxidative decarboxylation of α-ketoglutarate to produce succinate semialdehyde and CO_2_. The decarboxylation process is reliant on the cofactor of thiamine diphosphate. However, this enzyme’s biochemical and structural properties have not been well characterized. In this work, two α-ketoglutarate decarboxylases encoded by *MAE_06010* and *MiAbw_01735* genes from *Microcystis aeruginosa* NIES-843 (MaKGD) and NIES-4325 (MiKGD), respectively, were overexpressed and purified by using an *Escherichia coli* expression system. It was found that MaKGD exhibited 9.2-fold higher catalytic efficiency than MiKGD, which may be attributed to the absence of glutamate decarboxylase in *Microcystis aeruginosa* NIES-843. Further biochemical investigation of MaKGD demonstrated that it displayed optimum activity at pH 6.5–7.0 and was most activated by Mg^2+^. Additionally, MaKGD showed substrate specificity towards α-ketoglutarate. Structural modeling and autodocking results revealed that the active site of MaKGD contained a distinct binding pocket where α-ketoglutarate and thiamine diphosphate interacted with specific amino acid residues via hydrophobic interactions, hydrogen bonds and salt bridges. Furthermore, the mutagenesis study provided strong evidence supporting the importance of certain residues in the catalysis of MaKGD. These findings provide new insights into the structure-function relationships of α-ketoglutarate decarboxylases from cyanobacteria.

## 1. Introduction

Cyanobacteria, which are photosynthetic prokaryotes, play an essential role in the global carbon and nitrogen cycles due to their metabolic diversity and adaptability to various environments [1]. Within this group, *Microcystis aeruginosa* is found in lakes and other water bodies with high organic matter concentrations, often leading to water blooms. *Microcystis aeruginosa* NIES-843 is the first Microcystis with a fully sequenced genome consisting of 5,842,795 bp and contains 6312 protein-coding genes with a GC content of 42.3% [2].

**Figure 1:**
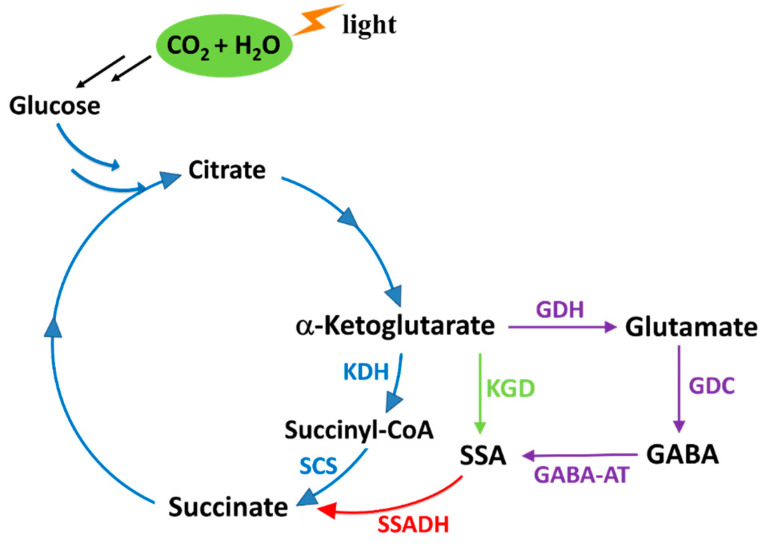
The tricarboxylic acid cycle bypasses of cyanobacteria. The KDH is absent in cyanobacteria. KDH: α-ketoglutarate dehydrogenase, SCS: succinyl-CoA synthase, GDH: glutamate dehydrogenase, GDC: glutamate decarboxylase, GABA-AT: γ-aminobutyric acid aminotransferase, KGD: α-ketoglutarate decarboxylase, SSADH: succinic semialdehyde dehydrogenase.

The tricarboxylic acid (TCA) cycle is essential for energy generation and biosynthesis in cyanobacteria (Figure 1). However, the conversion of α-ketoglutarate (α-KG) to succinic acid in the cyanobacterial TCA cycle differs from the canonical pathway, where α-KG is converted to succinic acid via succinyl-CoA by the function of α-KG dehydrogenase and succinyl-CoA synthetase (Figure 1). This conversion of α-KG to succinic acid in cyanobacteria occurs via the formation of succinic semialdehyde (SSA) through two known bypasses (Figure 1) [3]. The α-KG bypass involves two enzymes: α-ketoglutarate decarboxylase (KGD) and succinic semialdehyde dehydrogenase (SSADH). On the other hand, the γ-aminobutyric acid (GABA) shunt comprises three other enzymes, which are glutamate dehydrogenase (GDH), glutamate decarboxylase (GDC) and γ-aminobutyric acid aminotransferase (GABA-AT) (Figure 1).

KGD (EC 4.1.1.71) catalyzes the decarboxylation of α-KG to produce SSA and carbon dioxide, where thiamine diphosphate (ThDP) and divalent cations function as cofactors [4]. KGD belongs to the superfamily of α-ketoacids decarboxylases, which catalyze the non-oxidative decarboxylation of α-ketoacids to aldehydes [5]. Although other members of this superfamily, such as pyruvate decarboxylase, α-ketoisovalerate decarboxylase, indolepyruvate decarboxylase, and benzoylformate decarboxylase are well-characterized biochemically and structurally [6,7,8,9,10], less is known about the biochemical and structural properties of KGD. Nevertheless, its activity has been demonstrated in several organisms, including *Euglena gracilis* [11], *Leuconostoc oenos* [12] and *Bradyrhizobium japonicum* [13]. Further studies have revealed the dual functionality of the *MenD* gene in *Escherichia coli*, which encodes not only 2-succinyl-6-hydroxy-2,4-cyclohexadiene-1-carboxylic acid synthase, but also KGD [14]. Additionally, the protein encoded by the *Rv1248c* gene in *Mycobacterium tuberculosis* facilitates the decarboxylation of α-KG into a carbanion intermediate of α-KG bound to ThDP. Surprisingly, instead of producing SSA, this intermediate reacts with the carbonyl of glyoxylate to generate 2-hydroxy-3-oxoadipate [15]. However, none of the aforementioned studies have addressed the structure-function relationships of KGD, including identifying amino acid residues crucial for catalytic processes. 

The overexpression and biochemical characterization of KGDs from cyanobacteria of *Synechocystis* sp. PCC6803 (StKGD encoded by *sll1981* gene) and *Synechococcus* sp. PCC7002 (ScKGD encoded by *SYNPCC7002_A2770* gene) were previously reported by our laboratory [16,17]. It was observed that the activity of ScKGD was about 500-fold higher than that of StKGD with α-KG as a substrate, although these two enzymes shared over 80% amino acids sequences identities (Figure S1 and Table S1). Further investigations revealed that *Synechocystis* sp. 6803 featured a complete GABA shunt comprising all three enzymes, whereas the GABA shunt in *Synechococcus* sp. PCC7002 was incomplete due to the absence of GDC [3]. The relationship between the existence of GDC and the distinct activity levels of KGDs in these two strains remains unclear. Furthermore, our prior studies have not elucidated the structure-function correlations of KGD, thereby failing to unveil the pivotal amino acid residues implicated in the catalytic process.

In this study, two KGD proteins from *Microcystis aeruginosa* are overexpressed, purified and characterized. One KGD is encoded by the *MiAbW_01735* gene in *Microcystis aeruginosa* NIES-4325 (MiKGD hereafter), which has a complete GABA shunt. The other one is encoded by the *MAE_06010* gene in *Microcystis aeruginosa* NIES-843 (MaKGD hereafter), in which the GDC is absent, resulting in an incomplete GABA shunt. Due to its high activity, further investigation is being conducted on the biochemical properties and model structures of MaKGD. The results presented here might provide insights into the structure-function relationships of KGD enzymes from cyanobacteria. 

## 2. Results and Discussion

### 2.1. Overexpression, Purification and Native Mass Determination of Recombinant KGDs

**Figure 2:**
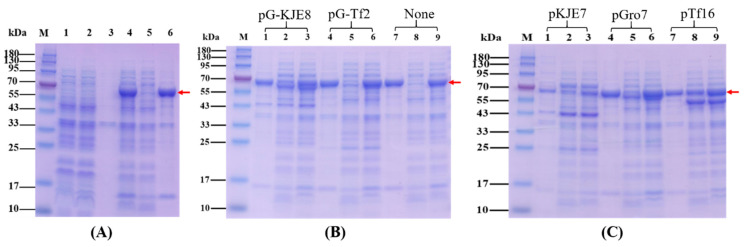
The overexpression of MaKGD. (**A**) The expression comparison of plasmids pET-28a and pET28a-*MAE_06010*. M: protein marker; Lanes 1–3: whole cell lysate, supernatant and cell pellet of plasmid pET-28a, respectively; Lanes 4–6: whole cell lysate, supernatant and cell pellet of pET28a-*MAE_06010*, respectively. (**B**,**C**) The effect of coexpression of chaperone plasmids and pET28a-*MAE_06010* on the soluble expression of MaKGD. M: protein marker; Lanes 1, 4 and 7: cell pellet; Lanes 2, 5 and 8: supernatant; Lanes 3, 6 and 9: whole cell lysate. The red arrow indicates MaKGD.

**Figure 3:**
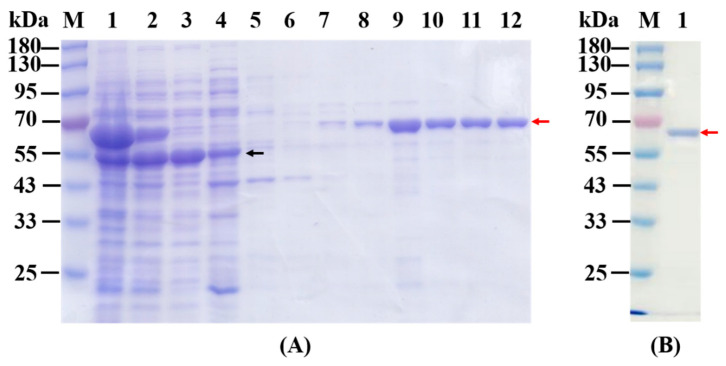
The purification of MaKGD. (**A**) The elution of MaKGD with various concentrations of imidazole. M: protein marker; Lanes 1–3: whole cell lysate, supernatant, and flow through, respectively; Lanes 4–8: 20, 40, 60, 80, and 100 mM imidazole elution, respectively; Lanes 9–12: 200 mM imidazole elution. (**B**) The purified MaKGD. The red arrow indicates MaKGD, black arrow indicates the protein overexpressed by the pTf16 chaperone plasmid.

The *E. coli* BL21(DE3) competent cells were transformed with pET28a-*MAE_06010* plasmid. The overexpression of MaKGD was checked by SDS-PAGE. The recombinant MaKGD protein comprises 550 amino acids, and the theoretical molecular weight is 59.82 kDa. As shown in Figure 2A, the dominant protein bands in lanes 4/6 were between 55 kDa and 70 kDa in molecular weight, which indicated that the MaKGD protein was overexpressed. However, there was no soluble MaKGD in the supernatant (lane 5 in Figure 2A). Meanwhile, the effect of temperature and concentration of isopropyl β-D-thiogalactoside (IPTG) on the solubility of MaKGD was checked. It was obvious that no soluble MaKGD was achieved when the plasmid of pET28a-*MAE_06010* was induced at various temperatures and concentrations of IPTG (Figure S2). Therefore, the effect of chaperone plasmids on the soluble expression of MaKGD was investigated (Figure 2B,C). Compared with the expression of pET28a-*MAE_06010* alone in *E. coli* BL21(DE3) (lanes 7–9 in Figure 2B), the coexpression of respective chaperone plasmids with pET28a-*MAE_06010* improved the soluble expression of MaKGD with varied potential (Figure 2B,C). Among them, chaperone plasmid pTf16 was the best one to promote the solubility of MaKGD protein (lanes 7–9 in Figure 2C). As a result, MaKGD protein was purified to be homogenous with the yield of 3.2 mg/g wet cells and the purity was estimated to be over 95% (Figure 3).

Chaperone plasmid pTf16 was also co-expressed with the plasmid pET28a-*MiAbW_01735* in *E. coli* BL21(DE3) cells. The theoretical molecular weight of MiKGD is also 59.82 kDa. The results showed that the solubility of MiKGD was significantly improved with the coexpression of the pTf16 chaperone plasmid (Figure S3). Similarly, recombinant MiKGD protein was purified and concentrated to the desired concentrations (Figure S3).

The native molecular weights of MaKGD and MiKGD are determined to be 242.26 kDa and 243.70 kDa, respectively, by the gel filtration method, as reported previously [16,18]. Based on these results, it can be inferred that both MaKGD and MiKGD exist as tetramers in their native states since their molecular weights of subunits are 59.82 kDa. It is consistent with the oligomer state of KGD from *Euglena gracilis*, which is also a tetramer [11]. The oligomer state of tetramer was observed in many α-ketoacid decarboxylases, such as benzoylformate decarboxylase from *Pseudomonas putida* [19] and pyruvate decarboxylases from various organisms [20]. 

### 2.2. Steady-State Kinetic Assay of KGDs Wild-Type

**Figure 4:**
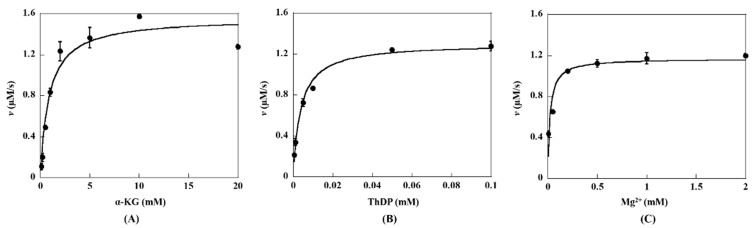
The kinetic profiles of MaKGD. (**A**) The initial rates as a function of α-KG concentrations, the concentrations of ThDP and Mg^2+^ were fixed at 1 mM and 2 mM, respectively. (**B**) The initial rates as a function of ThDP concentrations, the concentrations of α-KG and Mg^2+^ were fixed at 10 mM and 2 mM, respectively. (**C**) The initial rates as a function of Mg^2+^ concentrations, the concentrations of α-KG and ThDP were fixed at 10 mM and 1 mM, respectively.

The kinetic parameters of MaKGD were determined with α-KG, ThDP, and MgSO_4_ as substrates/cofactors (Figure 4). When the concentrations of ThDP and MgSO_4_ are fixed at 1 mM and 2 mM, respectively, the Michaelis constant (*K_M_*) value of α-KG is determined to be 0.87 ± 0.23 mM, and the maximum initial rate (*V*_max_) is 1.56 ± 0.10 μM/s (Figure 4A and Figure S4). The catalytic rate (*k*_cat_) and catalytic efficiency (*k*_cat_/*K_M_*) are calculated to be 7.8 ± 0.5 s^−1^ and 8.97 × 10^3^ M^−1^s^−1^, respectively, since the concentration of MaKGD is 0.2 μM in this assay (Table 1). Similarly, the *K*_0.5_ values of ThDP and Mg^2+^ are determined to be 0.0056 ± 0.0007 mM and 0.022 ± 0.001 mM, respectively (Figure 4B,C and Table 1). The concentrations of ThDP and Mg^2+^ are saturated for the kinetic determination of MaKGD with α-KG as substrate.

The catalytic efficiency of StKGD was extremely low in our previous study [17]. The possible reason was that the StKGD lost activity during the lengthy purification procedures [17]. In this study, StKGD was overexpressed with chaperone plasmid pTf16 and purified to be homogenous again. The kinetic parameters of StKGD are 3.83 ± 0.75 mM, 3.07 ± 0.42 s^−1^, and 0.80 × 10^3^ M^−1^s^−1^ for *K_M_*, *k*_cat_, and *k*_cat_/*K_M_*, respectively, with α-KG as substrate and saturated ThDP and Mg^2+^ as cofactors (Table 1). Nevertheless, the catalytic efficiency of StKGD is still only 8.9% of that of MaKGD.

The kinetic parameters of MiKGD are close to those of StKGD. The values of *K_M_*, *k*_cat_, and *k*_cat_/*K_M_* of MiKGD with α-KG as substrate are 4.47 ± 0.63 mM, 4.33 ± 0.37 s^−1^, and 0.97 × 10^3^ M^−1^s^−1^, respectively (Table 1). As shown in Table 1, the catalytic efficiencies of ScKGD and MaKGD are about 6- to 11-fold higher than those of StKGD and MiKGD. Considering the high degree of similarity in amino acid sequences and conserved active site residues among these KGDs (Figure S1 and Table S1), it is unclear why the KGDs from various sources display different activities. One hypothesis is that the activity of KGD is affected by the GABA shunt, which is known to be complete in *Synechocystis* sp. PCC6803 and *Microcystis aeruginosa* NIES-4325 [21,22]. However, the GABA shunt is broken in *Synechococcus* sp. PCC7002 and *Microcystis aeruginosa* NIES-843 due to the lack of GDC [3,21,22]. As a result, the KGDs from organisms that have the GABA shunt display lower activity compared to those that do not have the GABA shunt pathway, which relieves the metabolic burden of α-KG to SSA via glutamate and GABA leading to the low activity of KGD [3]. It is also true for the KGD from *Euglena gracilis* (EgKGD) with complete GABA shunt [23]. EgKGD had a catalytic efficiency of 0.31 × 10^3^ M^−1^s^−1^ [11,17], comparable to StKGD and MiKGD with complete GABA shunt. Due to its high catalytic activity, MaKGD was selected as the subject for further investigation regarding the detailed biochemical characterization and structural modeling of KGD from cyanobacteria *Microcystis aeruginosa*.

### 2.3. Effects of pH and Divalent Cations on Catalytic Activity of MaKGD

**Figure 5:**
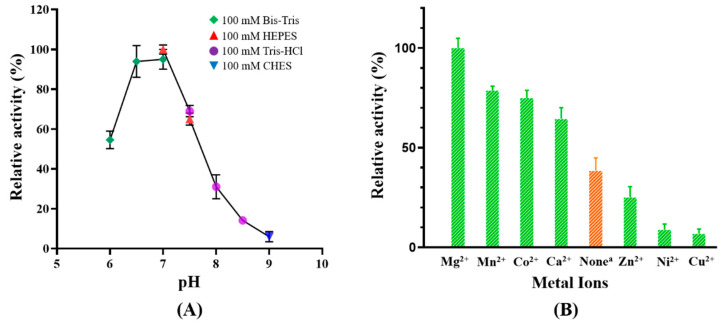
The effects of pH and divalent cations on the activity of MaKGD. (**A**) Effects of pH on the activity of MaKGD. The activity of MaKGD was designated as 100% at pH 7.0, 100 mM HEPES. (**B**) Effects of divalent cations on the activity of MaKGD. The activity of MaKGD with the addition of 2 mM Mg^2+^ was considered 100%. The concentrations of divalent cations are 2 mM in the assay buffer as described in Materials and Methods. ^a^: traces of metal ions may exist due to the purification procedure.

The optimal pH range for MaKGD is between 6.5–7.0, with a significant decline in activity observed above pH 8.0 and below pH 6.0 (Figure 5A). This optimal pH range for catalysis was also observed in other α-ketoacid decarboxylases, including ScKGD [16,24,25]. The optimal pHs of α-ketoacid decarboxylases are presumed to be associated with the pKa of the 4′-aminopyridinium group of the ThDP cofactor bound with enzymes [26,27]. Meanwhile, it was found that the subunit association equilibrium of α-ketoacid decarboxylases might be pH-dependent. For instance, the tetramer structure of indolepyruvate decarboxylase from *Enterobacter cloacae* was stabilized within the pH of 5.6–7.5 [9]. Hence, an alternate explanation for the observed optimal pH of MaKGD could be the stability of the tetramer structure of MaKGD within the pH range of 6.5–7.0. Furthermore, it was argued that the optimum pHs for α-ketoacid decarboxylases were probably related to the isoelectric point (pI) of the enzyme. The pIs of KGD from *Leuconostoc oenos* [12] and phenylpyruvate decarboxylase KDC4427 from *Enterobacter* sp. CGMCC 5087 [28] were found to be 4.2 and 5.73, respectively, and these two enzymes showed optimum activity at pH 5.3 and 6.5, respectively. The optimum pH is approximately one unit higher than the pI. The pI of MaKGD is 5.13, which might contribute to the optimum activity of MaKGD in weak acid conditions.

The effects of different divalent cations on the activity of MaKGD are shown in Figure 5B. The results show that the activity of MaKGD is dependent on metal ions. The best activator is Mg^2+^. Therefore, the activity of MaKGD is standardized by assigning a value of 100% when supplemented with 2 mM Mg^2+^ (Figure 5B). Including alternative divalent cations, such as Mn^2+^, Co^2+^, and Ca^2+^, results in 64–78% activity compared to Mg^2+^. It is noteworthy that MaKGD exhibits a substantial 38% activity even in the absence of metal ions (the orange bar in Figure 5B). The possible reason is that traces of metal ions may exist due to the purification procedure. Compared to the addition of Mg^2+^, the addition of Zn^2+^ results in a remarkable 75% decline in activity, whereas the addition of Ni^2+^ and Cu^2+^ substantially decreases the activity by approximately 90% (Figure 5B). These results indicate that Zn^2+^, Ni^2+^, and Cu^2+^ are good inhibitors of MaKGD. The extent to which divalent cations affect the activities of KGDs is variable and can differ depending on the specific cations. For instance, Co^2+^ inhibited the activity of KGD from *Leuconostoc oenos* by 50%, whereas Mg^2+^, Mn^2+^, Ca^2+^, or Ni^2+^ did not affect the activity [12]. When looking at KGD from *Euglena gracilis*, the activity was stimulated by both Mg^2+^ and Mn^2+^, with an increase of approximately 20%. However, Ca^2+^ and Co^2+^ did not affect the activity of EgKGD [11]. It has been proposed that the divalent cation (such as Mg^2+^) functions as an anchor by interacting with the phosphoryl group of ThDP through electrostatic bonds, thereby stabilizing the enzyme-cofactor complex and facilitating the decarboxylation reaction [5]. However, those ions which reduce or inhibit the activities of KGDs likely bind to the enzyme resulting in an inactive holoenzyme [8]. 

### 2.4. Determination of Substrate Specificity of MaKGD

The substrate specificity of MaKGD was investigated with various α-ketoacids substrates, including α-KG, pyruvate, 2-oxopentanoic acid, 3-methyl-2-oxobutanoic acid, 4-methyl-2-oxopentanoic acid and benzoylformate. The decarboxylation of α-ketoacids produces corresponding aldehydes, which are known to form a magenta-colored complex with Schiff’s reagent. As shown in Figure S5, the catalytic decarboxylation of α-KG by MaKGD produced an aldehyde which formed a magenta-colored complex with Schiff’s reagent (cuvette 2 in Figure S5A and cuvette 1 in Figure S5B). However, none of the reactions of pyruvate, 2-oxopentanoic acid, 3-methyl-2-oxobutanoic acid, 4-methyl-2-oxopentanoic acid or benzoylformate catalyzed by MaKGD led to the formation of aldehydes (cuvettes 2–6 in Figure S5B). Given the low detection limit of Schiff’s reagent [16], it is reasonable to conclude that MaKGD displays stringent substrate selectivity solely towards α-KG amongst the tested α-ketoacids.

Other KGDs, such as EgKGD and ScKGD, exhibited the same strict substrate specificity as MaKGD and did not catalyze the decarboxylation of pyruvate or 2-oxopentanoic acid to produce corresponding aldehydes [11,16]. This rigorous substrate specificity of KGDs is likely due to their physiological role in organisms lacking α-KG dehydrogenase, where the conversion of α-KG to succinic acid in the TCA cycle primarily relies on KGD and SSA [4,29]. 

Different from KGDs, other α-ketoacid decarboxylases exhibited a wider range of substrate specificity. For instance, the α-ketoisovalerate decarboxylase from *Lactococcus lactis* showed catalytic activity towards α-ketoisocaproate and α-ketomethylvalerate at 22.7% and 16.7% of that for α-ketoisovalerate, respectively [8]. Similarly, an α-ketoacid decarboxylase from *Proteus mirabilis* JN458 was capable of catalyzing the decarboxylation of various compounds, such as phenylpyruvate, 3-methyl-2-oxopentanoic acid, 3-methyl-2-oxobutanoic acid, indole-3-pyruvate, and pyruvate, with an activity range of 33.54% to 92.70% that of 4-methyl-2-oxopentanoic acid [25]. Additionally, the Aro10p protein from *Saccharomyces kudriavzevii* also displayed broad substrate specificity, showing comparable activity towards phenylpyruvate, 2-ketoisocaproate, 2-ketoisovalerate, 2-ketomethylvalerate, and 4-methylthio-2-oxobutanoic acid [30].

### 2.5. Structural Modeling and Autodocking of MaKGD with α-ketoacids

**Figure 6:**
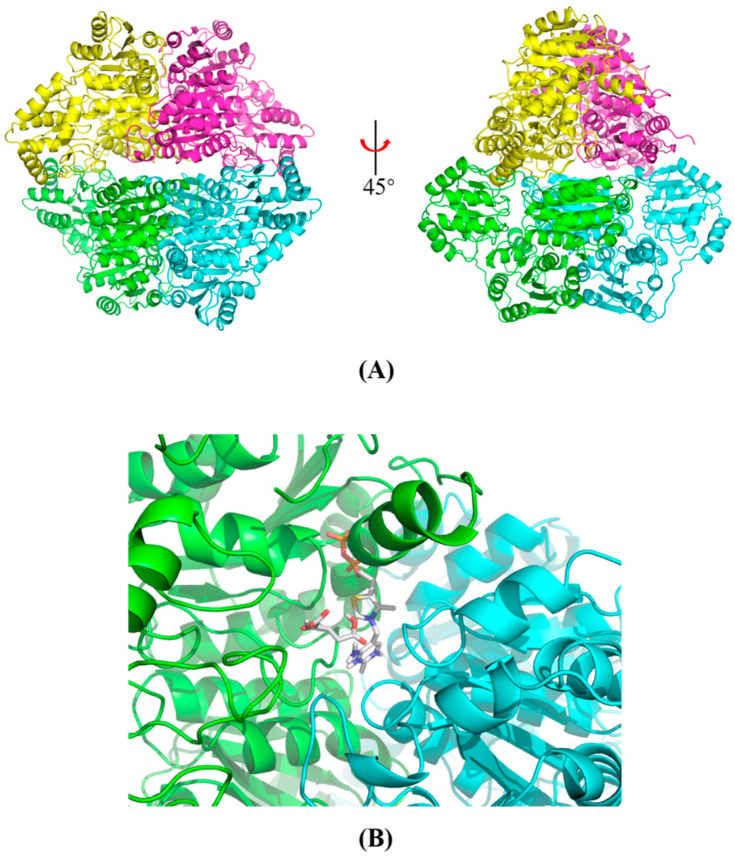
The cartoon structure and binding pocket of MaKGD. (**A**) Tetramer cartoon structure of MaKGD. (**B**) The close view of the binding pocket of MaKGD. The gray carbon sticks indicate α-KG and ThDP. The red, blue, yellow and orange colors indicate oxygen, nitrogen, sulfur and phosphorus atoms, respectively. Two subunits are colored green and cyan, respectively.

The initial crystal screenings of MaKGD were conducted using crystallization screen kits (MCSG I and II, Anatrace, OH, USA) with the sitting drop vapor diffusion method at 15 °C. However, no crystals were grown under these conditions. One possible reason for the difficulty in obtaining crystals was that this protein was prone to precipitation. Consequently, the tetrameric structure of MaKGD in its native state was calculated using AlphaFold (Figure 6 and File S1). The model structure of MaKGD was evaluated using the Ramachandran plot and Verify 3D (Figure S6). The Ramachandran plot showed that 90% of amino acid residues in the MaKGD model structure were located in the most favored regions, with only 0.2% found in the disallowed area (Figure S6A and Table S3). Additionally, the Verify 3D results indicated that 90% of the amino acid residues scored greater than or equal to 0.2 by the 3D–1D scoring function, confirming the validity of the MaKGD model structure (Figure S6B). These findings collectively demonstrated the reasonableness of the model structure of MaKGD.

**Figure 7:**
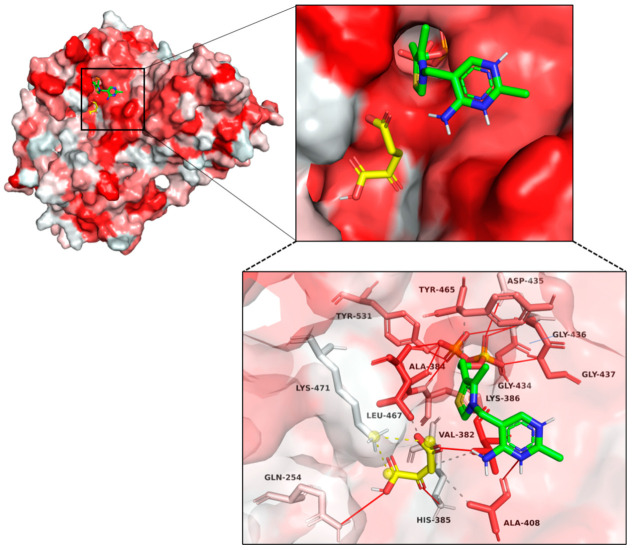
The autodocking and interactions of α-KG and ThDP with MaKGD. α-KG is indicated by yellow carbon sticks, and ThDP is indicated by green carbon sticks. The nitrogen atoms are colored blue. The specific residues of MaKGD are indicated by red to gray carbon sticks. The hydrophilicity of the protein surface is represented by a color scheme, with varying shades of red indicating the level of hydrophobicity. Darker shades correspond to higher levels of hydrophobicity. Solid red lines indicate hydrogen bonds, dashed gray lines indicate hydrophobic interactions and yellow dotted lines indicate salt bridges. Yellow spheres indicate charge centres.

The structure reveals that two subunits of MaKGD form a dimer, which then dimerizes to form a tetramer with a center-symmetric structure and an evident cavity between two dimers (Figure 6A). The autodocking results indicate that the substrate α-KG and the cofactor ThDP are located at the interface of two monomers (Figure 6B and File S2). Furthermore, the active site of MaKGD contains a distinct binding pocket where α-KG and ThDP interact with specific amino acid residues of MaKGD through hydrophobic interactions, hydrogen bonds, and salt bridges (Figure 7 and Figure S7). Specifically, α-KG interacts with Val382, Ala408, and Leu467 through hydrophobic interactions, while Gln254 and Lys386 form hydrogen bonds with α-KG, and Lys471 interacts with α-KG through salt bridge interaction. ThDP interacts with Tyr465 and His385 through hydrophobic and salt bridge interactions, respectively. Met410 also interacts with ThDP via hydrophobic interaction (Figure S7). Additionally, residues such as Ala384, His385, Ala408, Gly434, Asp435, Gly436, Gly437, Asp462, Leu467, and Tyr531 form multiple hydrogen bonds with ThDP (Figure S7). On the other hand, the autodocking results of various α-ketoacids and MaKGD reveal that these substances interact significantly less with the protein than α-KG, although the binding affinity of the cofactor ThDP is not significantly different (Figures S8–S12). This may explain why other α-ketoacids in Section 2.4 cannot be catalyzed by MaKGD.

### 2.6. Kinetic Characterization of MaKGD Variants

After autodocking of MaKGD with α-KG and ThDP, specific residues within the binding pocket were selected for mutation and subsequently characterized kinetically. The results are shown in Table 2. Leu467 engages in hydrophobic interactions with α-KG and forms hydrogen bonds with ThDP (Figure 7). Compared to the MaKGD wild-type, the L467A mutation did not considerably alter the *K_M_* of α-KG with MaKGD but decreased the *k*_cat_ by 2.9-fold. Interestingly, the L467A mutation increased the *K*_0.5_ of ThDP with MaKGD by 4.6-fold, hinting at the critical role of Leu467 in binding ThDP and maintaining the hydrophobic microenvironment. The *K_M_* of α-KG with the Y465A variant was measured to be 1.48 ± 0.27 mM, 1.7 times that of the MaKGD wild-type. In contrast, the *K*_0.5_ of ThDP with Y465A was 16 times that of the MaKGD wild-type at 0.090 ± 0.021 mM, which was consistent with the function of Tyr465 interacting with ThDP through hydrophobic interaction.

ThDP-dependent α-ketoacids decarboxylases have a conserved GDGX_25–30_N(C)N sequence for the binding of the pyrophosphate group of ThDP [20], where the Asp residue is crucial for the catalysis of the enzyme. The Asp435 residue in MaKGD is the Asp residue in the above-conserved sequence, which is then mutated to Glu, Ala and Asn, respectively. The D435A and D435N mutant proteins showed no detectable catalytic activity, while the D435E variant slightly increased to 120% of the MaKGD wild-type (Table 2). Asp462 participates in stabilizing ThDP via the hydrogen bond interaction. Then D462A, D462N and D462E mutants are obtained. The D462A mutant lacked activity, while the D462N maintained only 19% activity of wild-type enzyme due to an increased *K_M_* by 3-fold and a decreased *k*_cat_ by 5-fold. Spatially larger Asn residue disrupts substrate binding and active center interaction, greatly reducing enzyme activity. In addition, the *K_M_* (*K*_0.5_) values of α-KG and ThDP with the D462E variant were 2.38 ± 0.63 mM and 0.761 ± 0.143 mM, respectively, which were 2.7-fold and 136-fold higher than those of α-KG and ThDP with wild-type enzyme, respectively. Besides, Asp435 and Asp462 probably function as acid-base catalysts during catalysis. The *k*_cat_ of D435E and D462E was not significantly altered (Table 2). 

Meanwhile, the *K_M_* (*K*_0.5_) of α-KG and ThDP with the M410A variant were measured to be 0.53 ± 0.15 mM and 0.046 ± 0.010 mM, respectively (Table 2). The catalytic activity of M410A was only 17% of that of the MaKGD wild-type. Previous studies demonstrated that a strong hydrophobic residue was required to hold the “V” conformation of the ThDP cofactor [31]. For instance, replacing the ILE415 residue of pyruvate decarboxylase from yeast with amino acid residues possessing smaller side chains led to decreased activity, underscoring the importance of side chain size in maintaining the “V” conformation of ThDP [31]. Sequence alignment results show that the M410 residue in MaKGD corresponds to the I415 residue in pyruvate decarboxylase from yeast. The observation of low activity and the significant increase in *K*_0.5_ of ThDP with MaKGD suggest that the Met410 residue is essential for supporting the “V” configuration of the cofactor. The M410A mutagenesis effectively eliminates the hydrophobic interactions between Met410 and ThDP cofactor.

Another feature of ThDP-dependent α-ketoacids decarboxylases is the conservation of the pyrimidine-binding domain, where a Glu residue (Glu50 in MaKGD) forms a hydrogen bond with N-1 atom of the pyrimidine ring to regulate the ionization state of ThDP. The autodocking results have uncovered an expected hydrogen bond interaction between Glu50 from another subunit and ThDP (Figure S7C). Consequently, the E50A variant showed no activity (Table 2). The functional role of Glu50 residue in α-ketoacids decarboxylases has been studied thoroughly. For instance, the E50Q and E50N mutants of pyruvate decarboxylase from *Zymomonas mobilis* showed only 0.5% and 3% activity of the wild-type enzyme, respectively [32]. In addition, the activity of the E50Q mutant of pyruvate decarboxylase from yeast was reduced dramatically [33,34]. The Glu50 residue facilitates the equilibrium of the two tautomers, 4′-amino and 1′, 4′-imino forms of the pyrimidine ring of ThDP, thereby assisting in the rapid formation of the Ylide structure of ThDP for the subsequent reaction.

## 3. Materials and Methods

### 3.1. Materials

*MAE_06010* gene from *Microcystis aeruginosa* NIES-843 and *MiAbW_01735* gene from *Microcystis aeruginosa* NIES-4325 were optimized according to the codon usage in *E. coli* and synthesized by GENEWIZ (Suzhou, China). The chaperone plasmids set (pG-KJE8, pGro7, pKJE7, pG-Tf2, pTf16) was from Takara Bio Inc. (Dalian, China). *EasyPfu* DNA polymerase, T4 DNA ligase, DNA marker, Fast Mutagenesis System and *FlyCut* endonucleases were obtained from TransGen Biotech (Beijing, China). SSADH was purified by our laboratory [35,36]. The gene names used in this study were adopted from CyanoOmicsDB (http://www.cyanoomics.cn/lz/index, accessed on 25 September 2022) [22]. All other chemicals were purchased from Solarbio (Beijing, China) with the highest purity unless otherwise specified.

### 3.2. Cloning, Overexpression and Purification of MaKGD and MiKGD

The synthesized *MAE_06010* gene was cut by *NdeI* and *XhoI* endonucleases and then ligated with the pET28a vector, which was digested with the same two endonucleases to obtain the pET28a-*MAE_06010* plasmid (Figure S13). The plasmid of pET28a-*MiAbW_01735* (Figure S14) was constructed with the same method. Then, the *E. coli* BL21(DE3) competent cells were transformed with the constructed plasmid. The recombinant proteins encoded by the *MAE_06010* gene and *MiAbW_01735* gene were overexpressed and purified, respectively, by using the method described for the protein encoded by *SYNPCC7002_A2770* gene from *Synechococcus* sp. PCC7002 previously [16]. All the mutant proteins were overexpressed and purified with the same method as the wild-type by replacing the wild-type plasmid with mutated plasmids, which were constructed with the Fast Mutagenesis System using commercial primers (Table S2). 

### 3.3. Catalytic Activity Determination of MaKGD and MiKGD

The steady-state kinetics of KGD were determined according to the reported procedures at 25 °C [16]. Typically, the reaction mixture (200 μL) consisted of 1 mM ThDP, 2 mM NADP^+^, 2 mM Mg^2+^, 0.1–20 mM α-KG and a specific amount of MaKGD and SSADH in 100 mM HEPES, pH 7.0 buffer containing 10% glycerol. An appropriate amount of SSADH was used to ensure that the decarboxylation of α-KG to SSA was the rate-limiting step. The absorbance at 340 nm was recorded continuously by SpectraMax ABS (Molecular Devices, Shanghai, China). The initial reaction rates were determined by calculating the formation rate of NADPH at different concentrations of α-KG. The Michaelis-Menten equation fits the data to generate the maximum reaction rate (*V*_max_) and Michaelis constant (*K_M_*). The catalytic rate (*k*_cat_) was calculated by its definition of *V*_max_ over enzyme concentration. The *K*_0.5_ of ThDP was determined with fixed concentrations of α-KG and Mg^2+^ at 10 mM and 2 mM, respectively, and varied concentrations of ThDP at 0.002–0.1 mM. The *K*_0.5_ of Mg^2+^ as was determined with fixed concentrations of α-KG and ThDP at 10 mM and 1 mM, respectively, and varied concentrations of Mg^2+^ at 0.02–2 mM. The steady-state kinetic parameters of MiKGD were determined with the same protocols. All experiments were carried out triply.

### 3.4. Effects of pH on the Activity of MaKGD

The activity of MaKGD at different pH was determined by a two-step method. In the first step, the reaction mixture (200 μL) containing 1 mM ThDP, 2 mM Mg^2+^, 10 mM α-KG and 1.6 μM MaKGD in 100 mM buffers with pH of 6.0–9.0 was incubated at 25 °C for a specific time and then heated at 99.5 °C for 10 min to terminate the reaction. The buffers were Bis-Tris for pH 6.0, 6.5 and 7.0, HEPES for pH 7.0 and 7.5, Tris-HCl for pH 7.5, 8.0 and 8.5, and CHES for pH 9.0 containing 10% glycerol. In the second step, the produced SSA in the first step after a specific time was quantified by SSADH [36]. Briefly, the 20 μL aliquot after centrifugation from the first step was added to 2 mM NADP^+^, 2 mM Mg^2+^, 7 μM SSADH in 100 mM CHES, pH 9.5 buffer to make a 500 μL reaction mixture, whose absorbance was monitored at 340 nm. The amount of SSA was determined by calculating the amount of NADPH after the second step reaction was complete. Then, the activities of MaKGD under different pH could be calculated based on the produced SSA. 

### 3.5. Effects of Divalent Cations on the Activity of MaKGD

The effects of divalent cations on the activity of MαKGD were assayed by a two-step method. In the first step, the reaction mixture (200 μL) containing 1 mM ThDP, 10 mM α-KG, 1.6 μM MaKGD and 2 mM different divalent cations (MgSO_4_, MnCl_2_, CaCl_2_, CoCl_2_, ZnSO_4_, NiSO_4_ and CuSO_4_) in 100 mM HEPES, pH 7.0 buffer with 10% glycerol was incubated at 25 °C for a specific time and then heated at 99.5 °C for 10 min to terminate the reaction. The second step was to determine the amount of SSA produced in the first step with the same method as above. The activity of the reaction mixture without the addition of metal ions in the first step was used as a control.

### 3.6. Substrate Specificity Detection of MaKGD

The reaction mixture (1.5 mL) consisted of 1 mM ThDP, 2 mM Mg^2+^, 5 μM MaKGD and 10 mM various substrates (α-KG, pyruvate, 2-oxopentanoic acid, 3-methyl-2-oxobutanoic acid, 4-methyl-2-oxopentanoic acid or benzoylformate) in 100 mM HEPES, pH 7.0 with 10% glycerol. After being incubated at 25 °C for 5 h, the reaction mixture was heated at 99.5 °C for 10 min and centrifugated, and then the supernatant aliquot (500 μL) was mixed with Schiff’s reagent at the ratio of 1:3 to check the solution color. The reaction mixtures without MaKGD and with heating-deactivated α-KGD were used as control.

### 3.7. Structural Modeling, Model Validation and Autodocking of MaKGD with α-ketoacids

The model structure of MaKGD was predicted by AlphaFold [37]. The validation of the MaKGD model structure was carried out using the method described in our previous report [18]. The protein-ligand/substrate docking was completed by AutoDockTools-1.5.7 [38]. The protein docking center coordinates x = 5.029, y = 8.962, z = 7.991 was used with a grid spacing of 0.375 Å and a docking box of size 15 Å × 15 Å × 15 Å was set up based on the spatial structure of ThDP located in benzoylformate decarboxylase from *Pseudomonas putida* (Protein Data Bank ID: 1BFD). The protein-ligand interaction forces were analyzed using the PLIP online website (https://plip-tool.biotec.tu-dresden.de/plip-web/plip/index, accessed on 25 May 2023). The model structures were visualized by the software of PyMOL 1.3.x.

## 4. Conclusions

In this study, the biochemical characterization of recombinant MaKGD and MiKGD from *Microcystis aeruginosa* NIES-843 and NIES-4325, respectively, was performed for the first time. The kinetic analysis revealed that MaKGD exhibited a 9.2-fold higher catalytic efficiency than MiKGD. Further investigation demonstrated that MaKGD displayed optimal activity at pH 6.5–7.0, and among the tested divalent cations, Mg^2+^ was the most effective activator. Additionally, MaKGD exhibited strict substrate specificity towards α-KG. Structural modeling and autodocking analyses revealed that several residues in the binding pocket of MaKGD interacted with α-KG and ThDP via hydrophobic interactions, hydrogen bonds, and salt bridges.

## Figures and Tables

**Table 1:** The steady-state kinetic parameters of KGDs from different sources.

Proteins	α-KG			ThDP	Mg^2+^
	*K_M_* (mM)	*k*_cat_ (s^−1^)	*k*_cat_/*K_M_* (M^−1^s^−1^)	*K*_0.5_ (mM) ^a^	*K*_0.5_ (mM) ^b^
StKGD [17]	21 ± 4	0.26 ± 0.03	12.38	0.078 ± 0.028	Not available
StKGD ^c^	3.83 ± 0.75	3.07 ± 0.42	0.80 × 10^3^	0.063 ± 0.019	0.080 ± 0.007
ScKGD [16]	0.19 ± 0.02	1.20 ± 0.32	6.32 × 10^3^	0.031 ± 0.004	0.059 ± 0.005
MaKGD	0.87 ± 0.23	7.80 ± 0.50	8.97 × 10^3^	0.0056 ± 0.0007	0.022 ± 0.003
MiKGD	4.47 ± 0.63	4.33 ± 0.37	0.97 × 10^3^	0.026 ± 0.006	0.037 ± 0.004

^a^: the concentration of ThDP for half-saturation of activity. ^b^: the concentration of Mg^2+^ for half-activation of the enzyme. ^c^: StKGD was purified from the coexpression with chaperone plasmid pTf16.

**Table 2:** The steady-state kinetic parameters of MaKGD wild-type and variants.

Proteins	α-KG		Relative Catalytic Rate	ThDP
	*K_M_* (mM)	*k*_cat_ (s^−1^)	(% of Wild-Type)	*K*_0.5_ (mM)
wild-type	0.87 ± 0.23	7.80 ± 0.50	100	0.0056 ± 0.0007
L467A	1.22 ± 0.51	2.70 ± 0.32	35	0.026 ± 0.004
Y465A	1.48 ± 0.27	1.23 ± 0.25	15	0.090 ± 0.021
D435E	1.74 ± 0.60	9.73 ± 0.63	120	0.041 ± 0.003
D435A	NA	NA	NA	NA
D435N	NA	NA	NA	NA
D462E	2.38 ± 0.63	9.00 ± 0.47	115	0.761 ± 0.143
D462N	3.92 ± 0.45	1.50 ± 0.36	19	0.008 ± 0.001
D462A	NA	NA	NA	NA
M410A	0.53 ± 0.15	1.33 ± 0.22	17	0.046 ± 0.010
E50A	NA	NA	NA	NA

NA: No activity.

## Data Availability

The data presented in this study are available in the manuscript and the Supplementary Materials.

## Supplementary Materials

The following supporting information can be downloaded at: https://www.mdpi.com/article/10.3390/ijms241512198/s1.
