# Supplementary material for: Biochemical and Structural Insights into a Thiamine Diphosphate-Dependent α-Ketoglutarate Decarboxylase from Cyanobacterium Microcystis aeruginosa NIES-843
Source: Int J Mol Sci. 2023 Jul 30;24(15):12198. doi: 10.3390/ijms241512198 (PMC10418658; doi:10.3390/ijms241512198)
Supplement: Supplementary file 1 [file ijms-24-12198-s001.zip › ijms-2502851-supplementary-revised.pdf]

Biochemical and Structural insights into a Thiamine Diphosphate-dependent α-ketoglutarate decarboxylase from Cyanobacterium *Microcystis aeruginosa* NIES-843

Zhi-Min Li[1, #], Ziwei Hu[2, #], Xiaoqin Wang[2, §], Suhang Chen[2], Weiyan Yu[2], Jianping Liu[2, *] and Zhimin Li[2, 3, *]

[1] College of Chemistry and Materials, Jiangxi Agricultural University, Nanchang 330045, China

[2] College of Bioscience and Bioengineering, Jiangxi Engineering Laboratory for the Development and Utilization of Agricultural Microbial Resources, Jiangxi Agricultural University, Nanchang 330045, China

[3] Collaborative Innovation Center of Postharvest Key Technology and Quality Safety of Fruits and Vegetables in Jiangxi Province, Jiangxi Agricultural University, Nanchang 330045, China

*Correspondence: jianpingliu@jxau.edu.cn (J.L.); zhiminli@jxau.edu.cn (ZL)

[#] These authors contributed equally to this work.

[§] Current address: National Key Laboratory of Bioreactor Engineering, East China University of Science and Technology, Shanghai 200237, China

File S1: Coordinates of the apo model structure of MaKGD.

File S2: Coordinates of the complex model structure of MaKGD with ThDP/α-KG.

In File S2, the α-KG ligand is labelled as UNL and ThDP cofactor is labelled as residue 530 (TPP).

Table S1. Amino acids sequences identities of α-ketoglutarate decarboxylases from various cyanobacteria

Identities (%)	StKGD	ScKGD	CtKGD	MaKGD	MiKGD
StKGD	100				
ScKGD	80.00	100			
CtKGD	85.64	81.83	100		
MaKGD	83.45	77.25	84.36	100	
MiKGD	83.82	77.80	85.27	97.82	100

StKGD: α-ketoglutarate decarboxylase encoded by *sll1981* gene from *Synechocystis* sp. PCC6803.

ScKGD: α-ketoglutarate decarboxylase encoded by *SYNPCC7002_A2770* gene from *Synechococcus* sp. PCC7002.

CtKGD: α-ketoglutarate decarboxylase encoded by *cce4227* gene from *Cyanothece* sp. ATCC51142

MaKGD: α-ketoglutarate decarboxylase encoded by *MAE_06010* gene from *Microcystis aeruginosa* NIES-843.

MiKGD: α-ketoglutarate decarboxylase encoded by *MiAbW_01735* gene from *Microcystis aeruginosa* NIES-4325.

Table S2. Primer sequences of MaKGD mutants

Mutants	Primers	Primer Sequences
E50A	E50A-F	ATTACCACCCGCCATG***CG***CAAGGCGCGG
	E50A-R	***CG***CATGGCGGGTGGTAATAAATTTAATGC
D435A	D435A-F	ATTGTGGCGGTGACCGGCG***CG***GGCGGCTTTAT
	D435A-R	***CG***CGCCGGTCACCGCCACAATGCGTTTGTTC
D435E	D435E-F	ATTGTGGCGGTGACCGGCGA***A***GGCGGCTTTAT
	D435E-R	***T***TCGCCGGTCACCGCCACAATGCGTTTGTTC
D435N	D435N-F	ATTGTGGCGGTGACCGGC***A***A***C***GGCGGCTTTATG
	D435N-R	***G***T***T***GCCGGTCACCGCCACAATGCGTTTGTTC
M410A	M410A-F	AACGGCTTCGCCGCG***GC***GGGCATTGCGATT
	M410A-R	***GC***CGCGGCGAAGCCGTTGCTAATAATG
Y465A	Y465A-F	TAACGATGGCGGC***GCG***GGCCTGATTGA
	Y465A-R	***CGC***GCCGCCATCGTTAAAAATCAGGGTC
L467A	L467A-F	GATGGCGGCTATGGC***GC***GATTGAATGG
	L467A-R	***GC***GCCATAGCCGCCATCGTTAAAAATCAGG
D462A	D462A-F	GACCCTGATTTTTAACG***CG***GGCGGCTATGG
	D462A-R	***CG***CGTTAAAAATCAGGGTCACAAACGG
D462E	D462E-F	GACCCTGATTTTTAACGA***A***GGCGGCTATG
	D462E-R	***T***TCGTTAAAAATCAGGGTCACAAACGGGG
D462N	D462N-F	GACCCTGATTTTTAAC***A***A***C***GGCGGCTATG
	D462N-R	***G***T***T***GTTAAAAATCAGGGTCACAAACGG

Note: Mutation sites are marked in red italic bold.

Table S3. Ramachandran plot statistics of MaKGD model structure computed with PROCHECK program

Residue regions	Proportion (%)
residues in most favored regions	90.0
residues in additional allowed regions	8.44
residues in generously allowed regions	1.34
residues in disallowed regions	0.22
non-glycine and non-proline residues	100.0

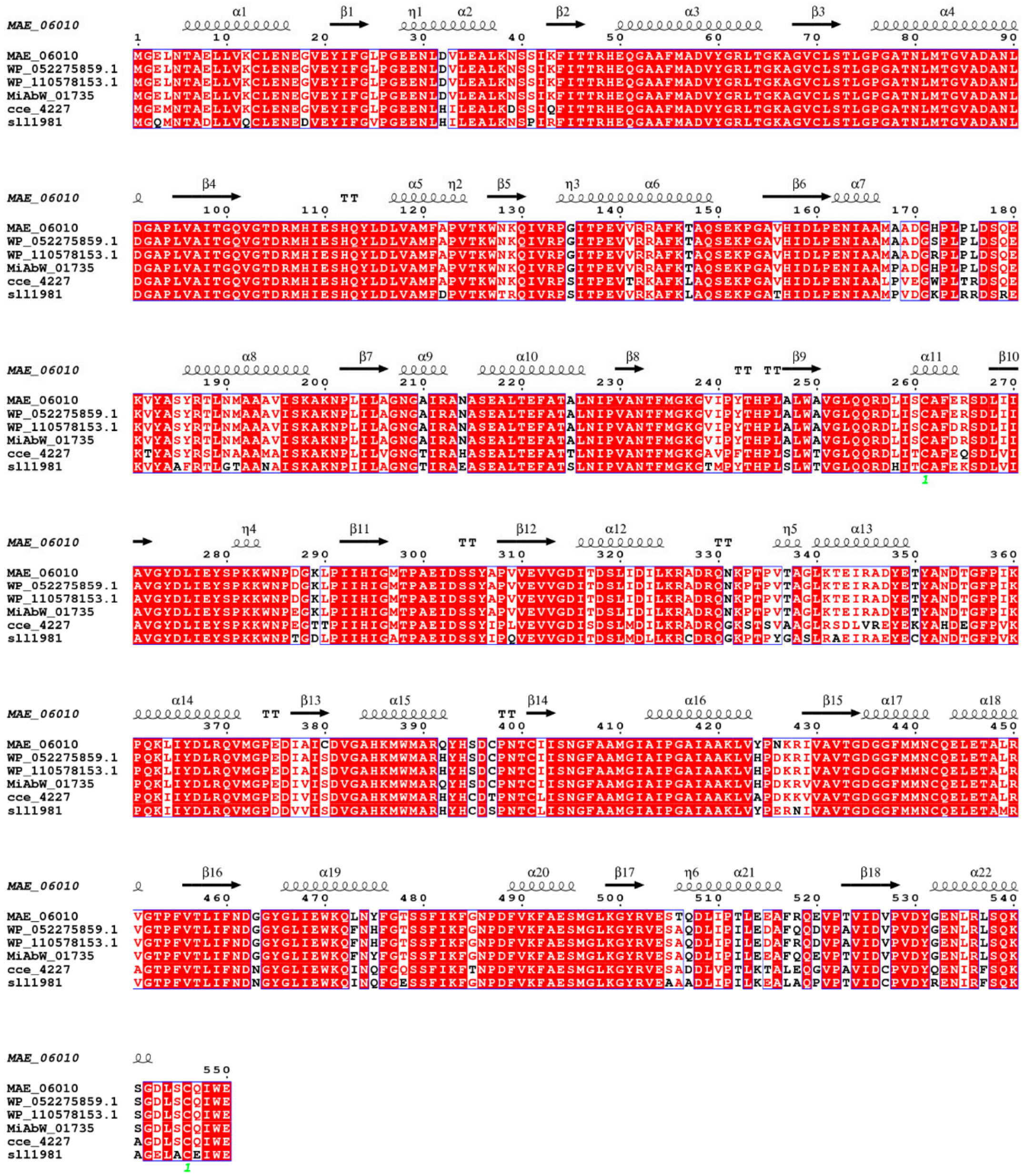

Figure S1. The multiple sequences alignment of KGDs from six different cyanobacteria. MAE_06010: MaKGD from *Microcystis aeruginosa* NIES-843. WP_052275859.1 and WP_110578153.1: two acetolactate synthases large subunit from *Microcystis aeruginosa*. MiAbW_01735: MiKGD from *Microcystis aeruginosa* NIES-4325. cce_4227: CtKGD from *Cyanothece* sp. ATCC51142. sll1981: StKGD from *Synechocystis* sp. PCC6803. The green number 1 indicates the possible disulfide bond.

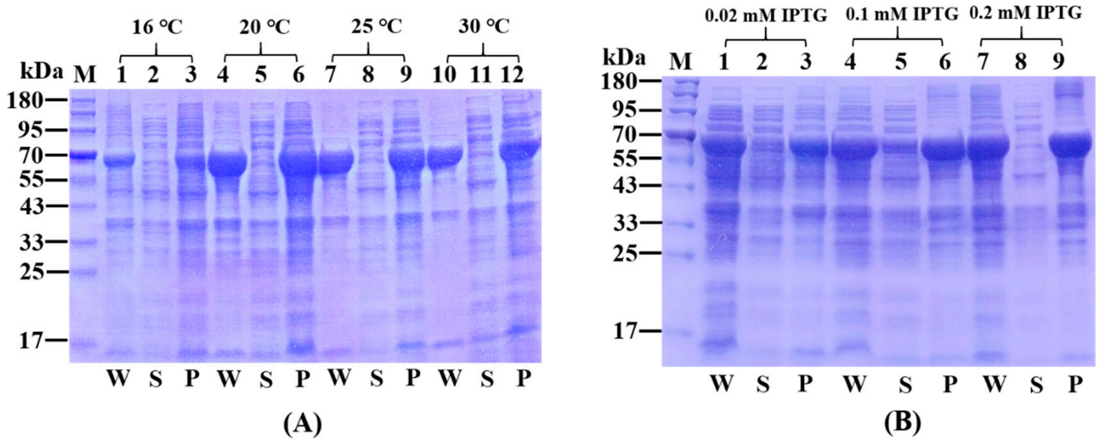

Figure S2. The effects of temperature and IPTG concentrations on the expression of MaKGD. W: whole cell lysate, S: supernatant, P: cell pellet.

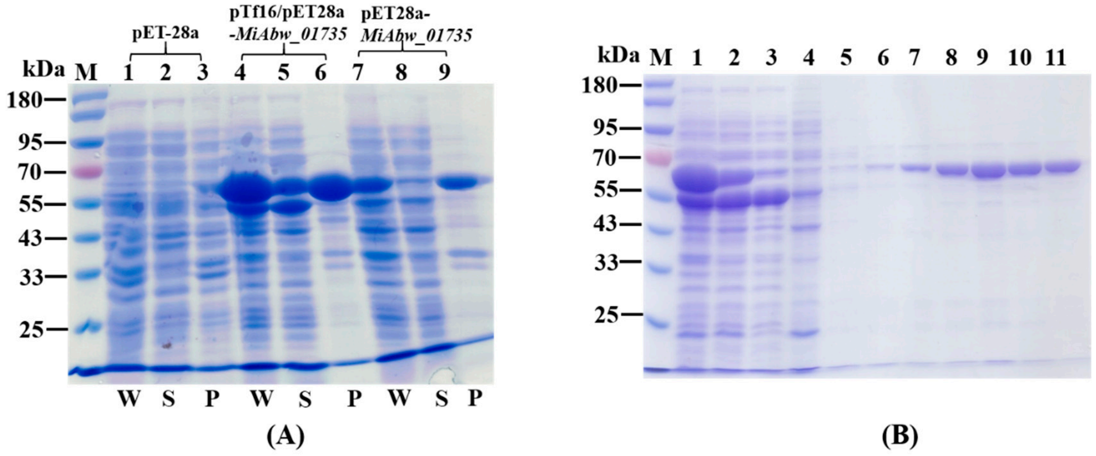

Figure S3. Expression and purification of MiKGD. (A) The overexpression of MiKGD. W: whole cell lysate, S: supernatant, P: cell pellet. (B) The purification of MiKGD. M: protein marker; Lanes 1-3: whole cell lysate, supernatant and flow through, respectively; Lanes 4-8: 20, 40, 60, 80, and 100 mM imidazole elution, respectively; Lanes 9-11: 200 mM imidazole elution.

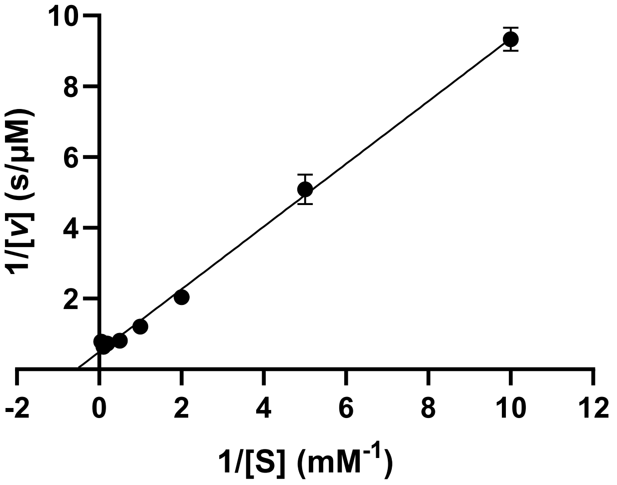

Figure S4. Lineweaver-Burk plot. The reciprocal initial rates as function of reciprocal α-KG concentrations, the concentrations of ThDP and Mg^{2+} were fixed at 1 mM and 2 mM, respectively.

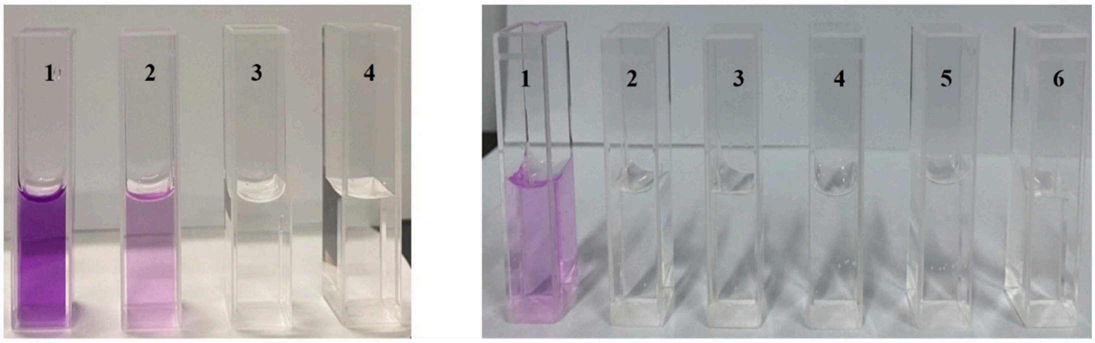

(A) **(B)**

Figure S5. Detection of aldehydes with Schiff's reagent. (A) Verification of Schiff's reagent. The key substances in cuvettes 1-4 are SSA, α-KG with MaKGD, α-KG with deactivated MaKGD, and α-KG alone, respectively. (B) Detection of products of α-ketoacids catalyzed by MaKGD. The α-ketoacids in cuvettes 1-6 are α-KG, pyruvate, 2-oxopentanoic acid, 3-methyl-2-oxobutanoic acid, 4-methyl-2-oxopentanoic acid, and benzoylformate, respectively. The reaction mixture (1.5 mL) consisted of 1 mM ThDP, 2 mM Mg^{2+}, 5 μM MaKGD and 10 mM various substrates (α-KG, pyruvate, 2-oxopentanoic acid, 3-methyl-2-oxobutanoic acid, 4-methyl-2-oxopentanoic acid or benzoylformate) in 100 mM HEPES, pH 7.0 with 10% glycerol. The mixture was incubated at 25 °C for 5 h, and then the produced aldehyde was detected by Schiff's reagent.

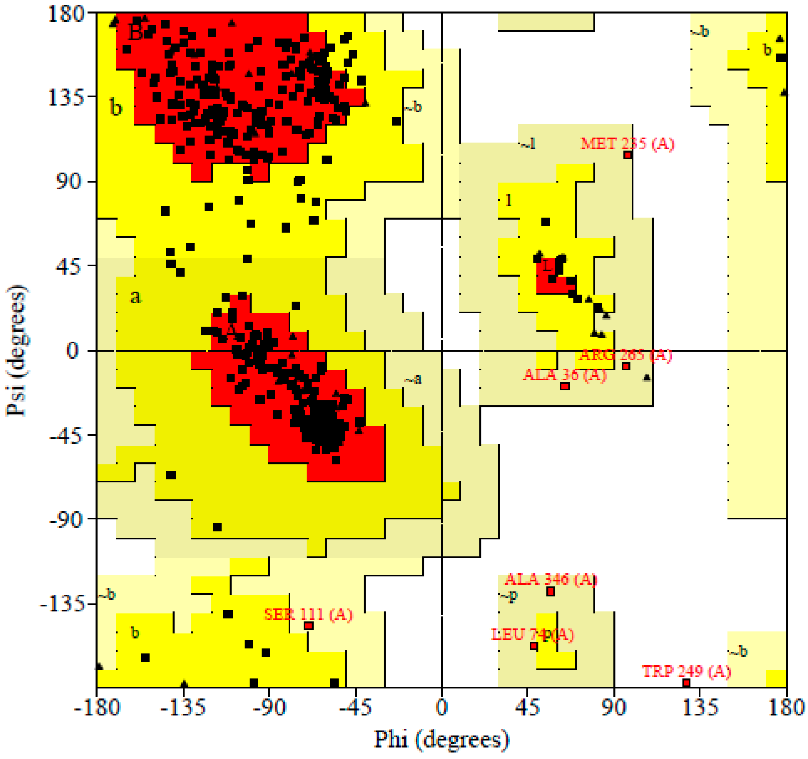

(A)

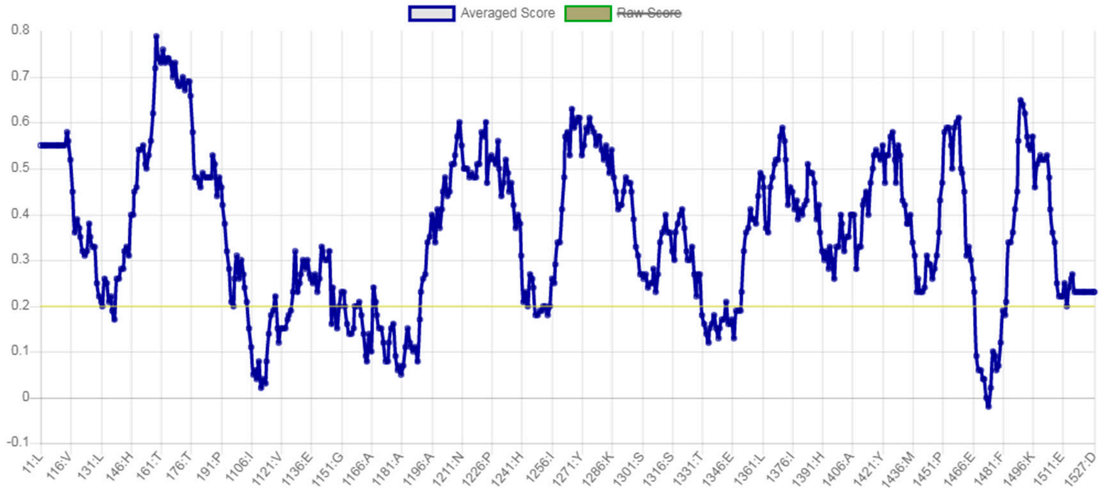

(B)

Figure S6. The validation of MaKGD model structure. (A) Ramachandran Plot of MaKGD model structure. (B) Verify 3D scores of MaKGD model structure.

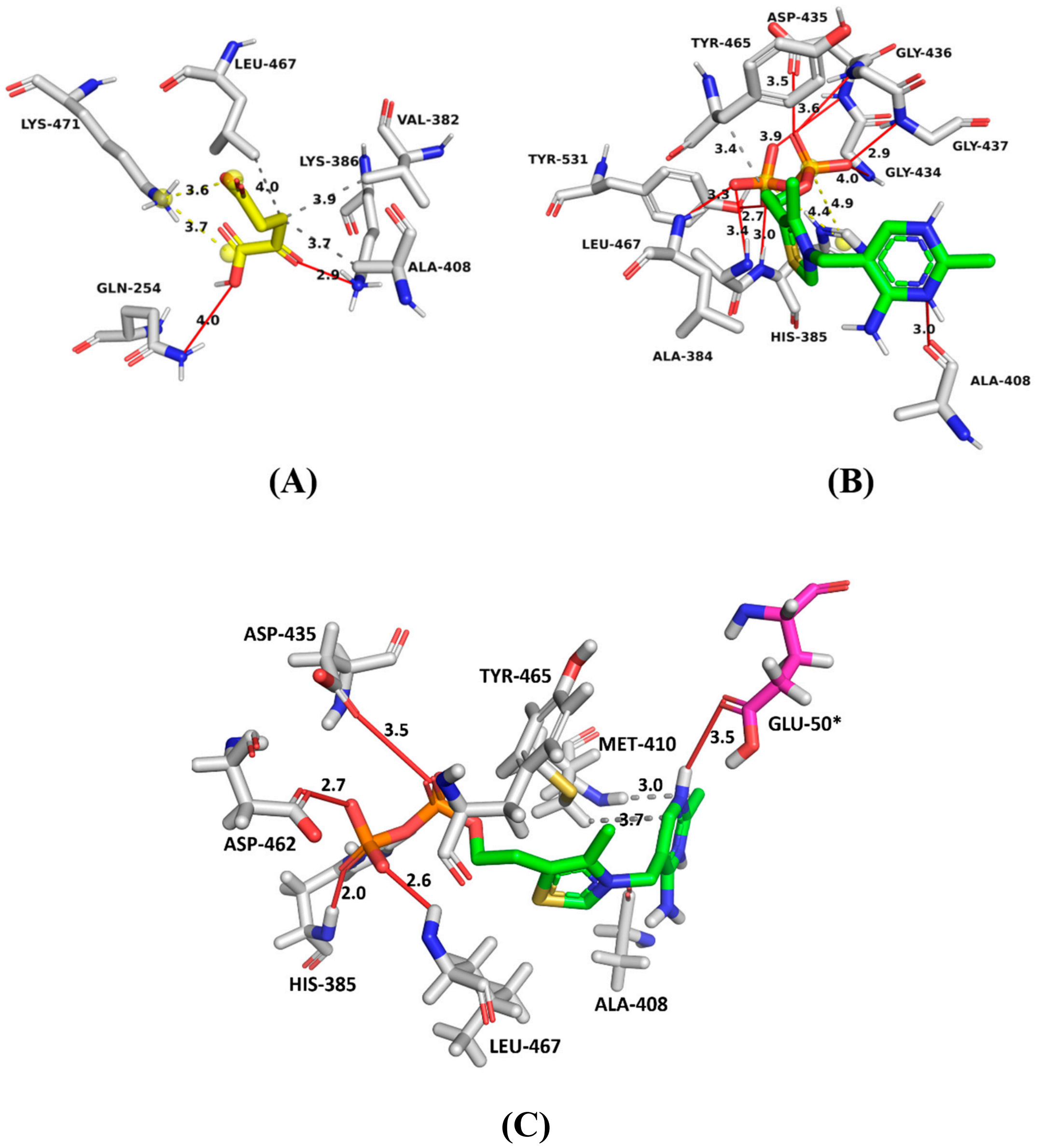

Figure S7. The interactions between substrate α-KG (A) and cofactor ThDP (B) with specific residues of MaKGD. α-KG is indicated by yellow carbon sticks and ThDP is indicated by green carbon sticks. (C) Close view of residues around cofactor ThDP. The specific residues of MaKGD are indicated by gray carbon sticks. The GLU-50* with magenta sticks is from another subunit. Solid red lines indicate hydrogen bonds, dashed gray lines indicate hydrophobic interactions, and yellow dotted lines indicate salt bridges. Yellow spheres indicate charge centers.

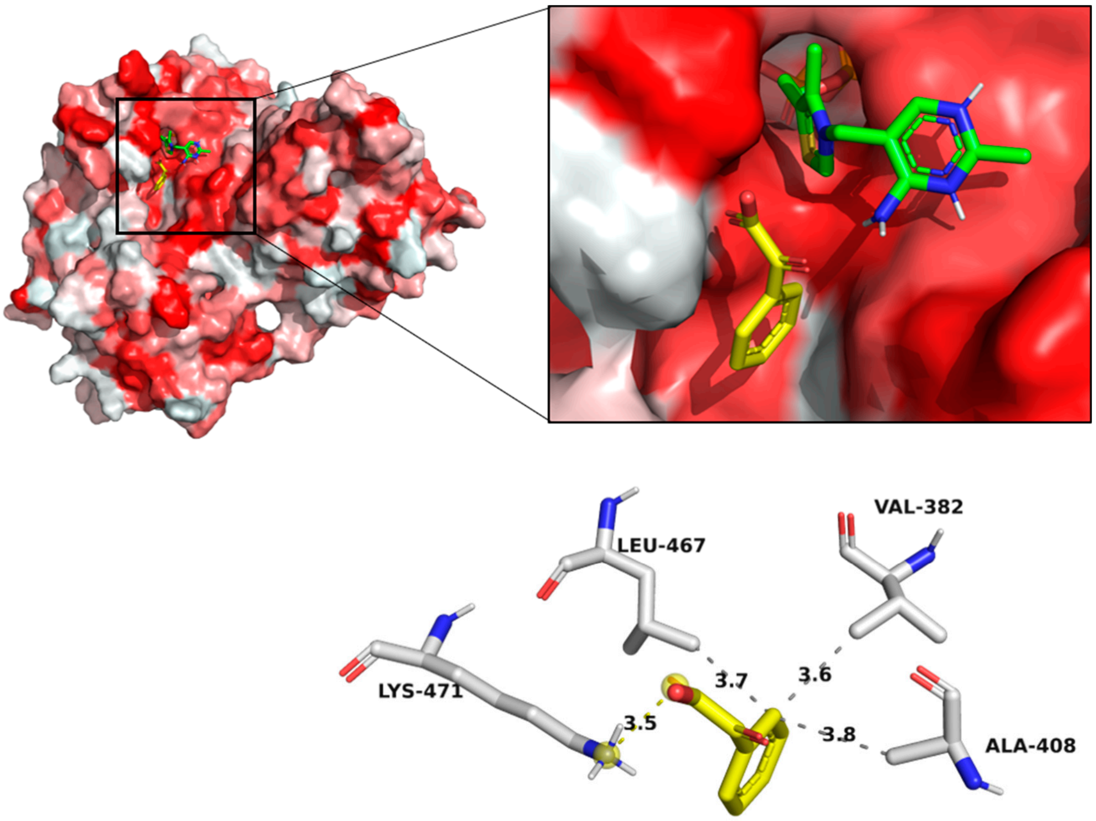

Figure S8. The autodocking and interactions of benzoylformate with MaKGD. Benzoylformate is indicated by yellow carbon sticks and ThDP is indicated by green carbon sticks. The specific residues of MaKGD are indicated by gray carbon sticks. Dashed gray lines indicate hydrophobic interactions, and yellow dotted lines indicate salt bridges. Yellow spheres indicate charge centers.

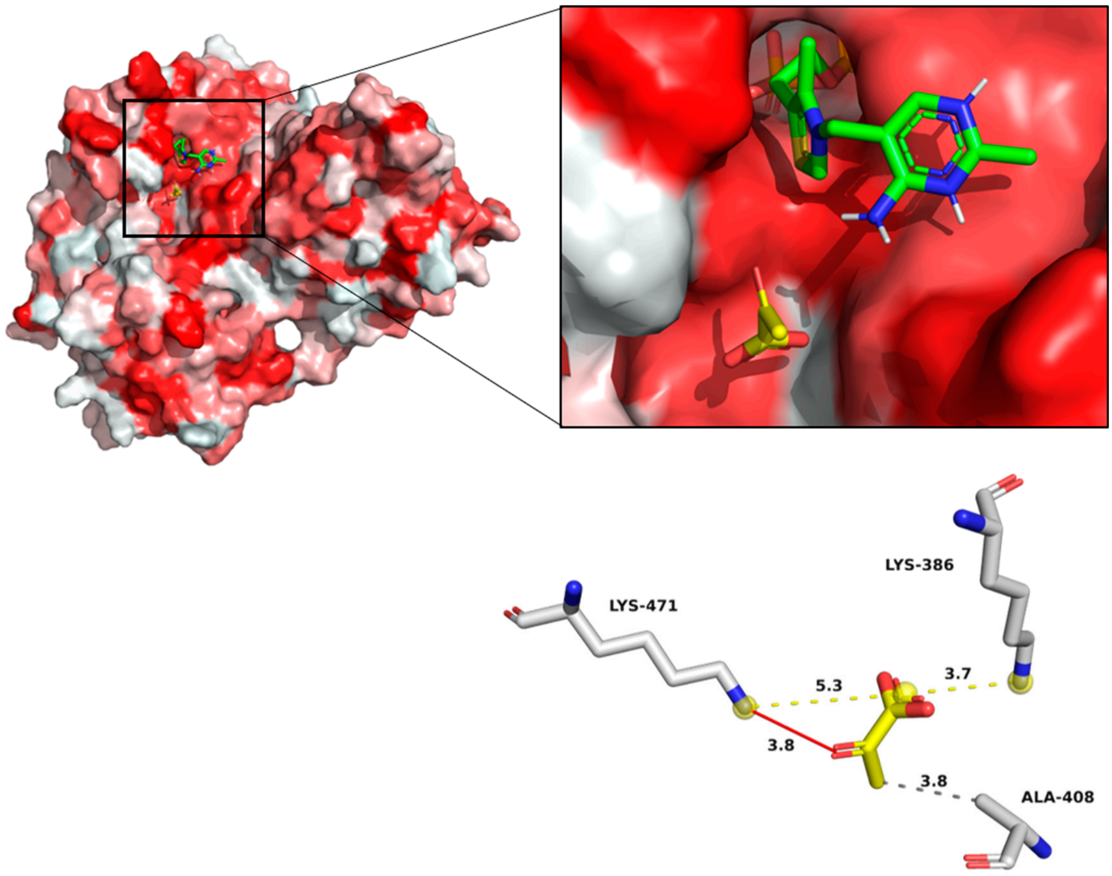

Figure S9. The autodocking and interactions of pyruvate with MaKGD. Pyruvate is indicated by yellow carbon sticks and ThDP is indicated by green carbon sticks. The specific residues of MaKGD are indicated by gray carbon sticks. Solid red lines indicate hydrogen bonds, dashed gray lines indicate hydrophobic interactions, and yellow dotted lines indicate salt bridges. Yellow spheres indicate charge centers.

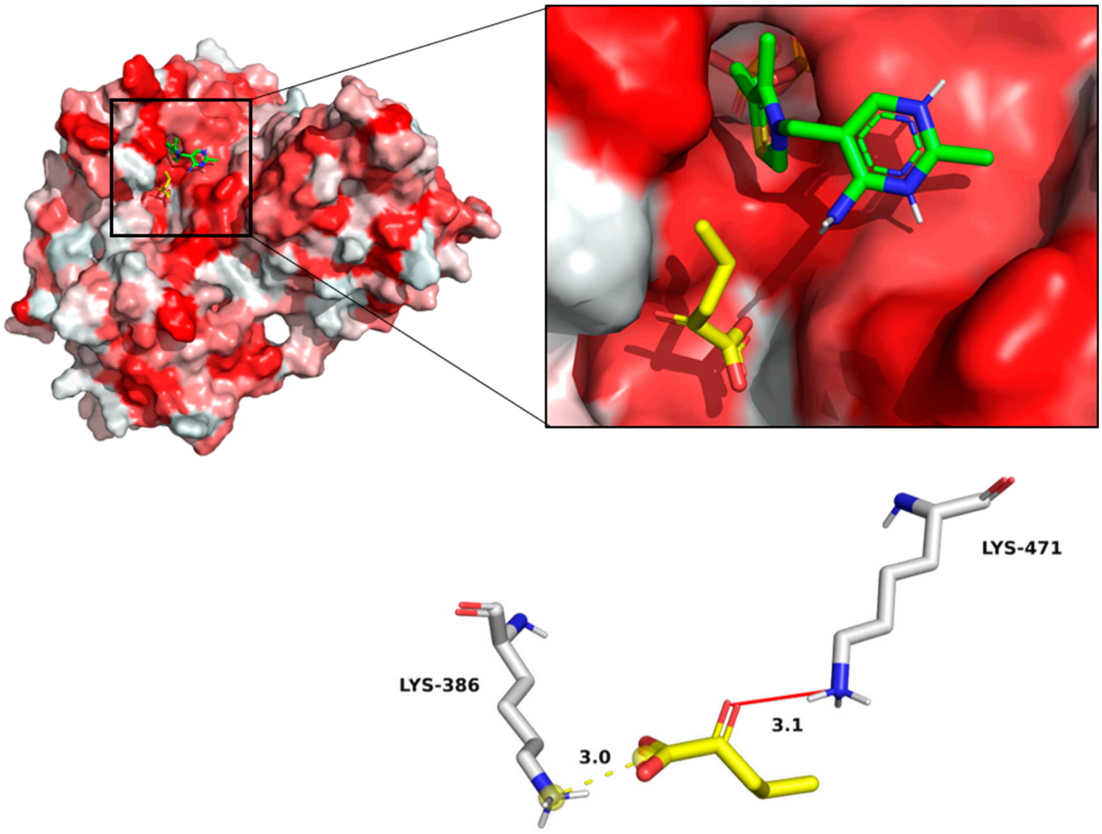

Figure S10. The autodocking and interactions of 2-oxopentanoic acid with MaKGD. 2-oxopentanoic acid is indicated by yellow carbon sticks and ThDP is indicated by green carbon sticks. The specific residues of MaKGD are indicated by gray carbon sticks. Solid red lines indicate hydrogen bonds, and yellow dotted lines indicate salt bridges. Yellow spheres indicate charge centers.

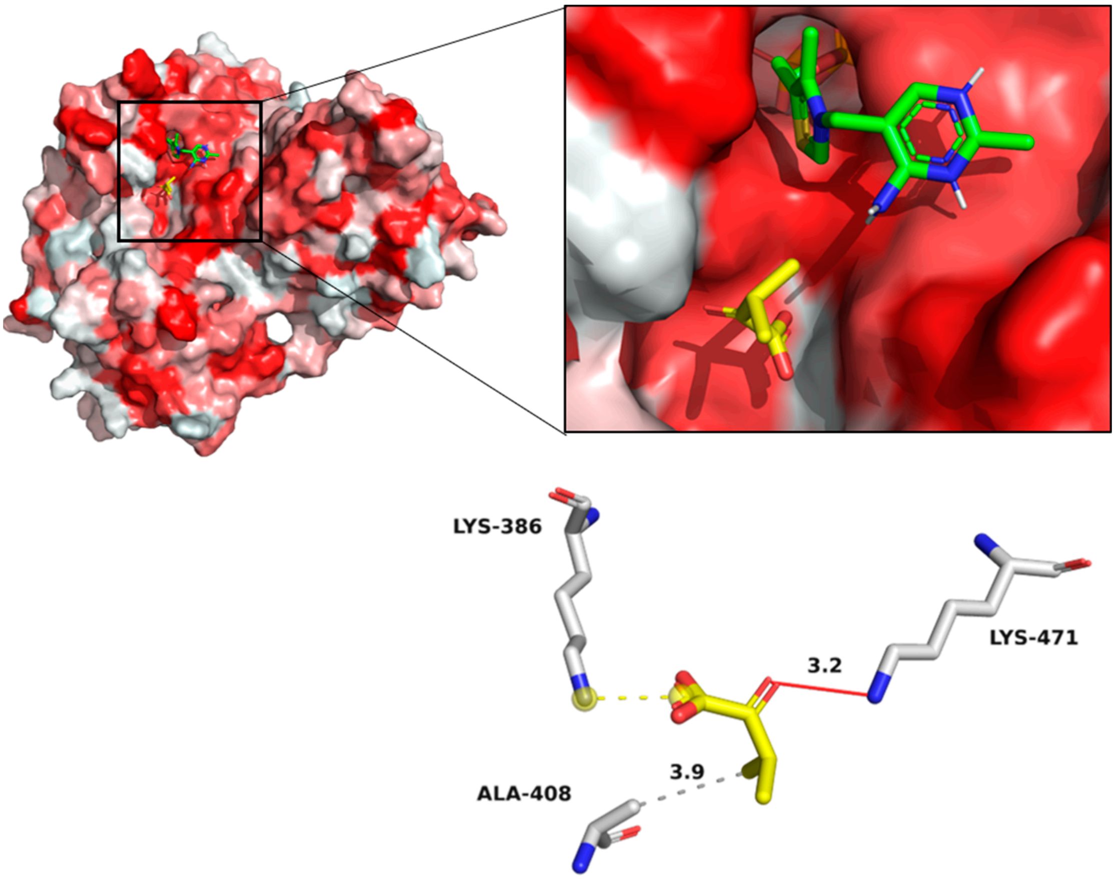

Figure S11. The autodocking and interactions of 3-methyl-2-oxobutanoic acid with MaKGD. 3-methyl-2-oxobutanoic acid is indicated by yellow carbon sticks and ThDP is indicated by green carbon sticks. The specific residues of MaKGD are indicated by gray carbon sticks. Solid red lines indicate hydrogen bonds, dashed gray lines indicate hydrophobic interactions, and yellow dotted lines indicate salt bridges. Yellow spheres indicate charge centers.

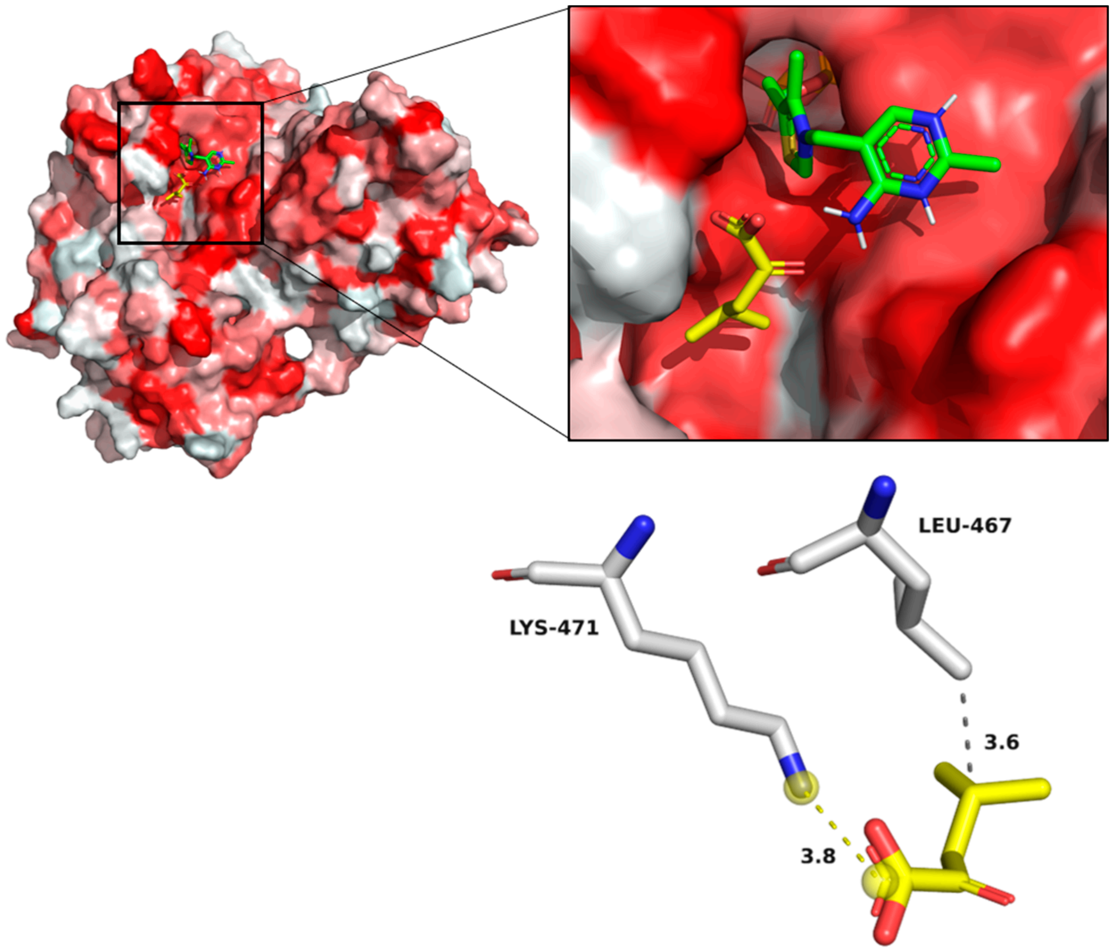

Figure S12. The autodocking and interactions of 4-methyl-2-oxopentanoic acid with MaKGD. 4-methyl-2-oxopentanoic acid is indicated by yellow carbon sticks and ThDP is indicated by green carbon sticks. The specific residues of MaKGD are indicated by gray carbon sticks. Gray dotted lines indicate salt bridges, and yellow dotted lines indicate hydrophobic interactions. Yellow spheres indicate charge centers.

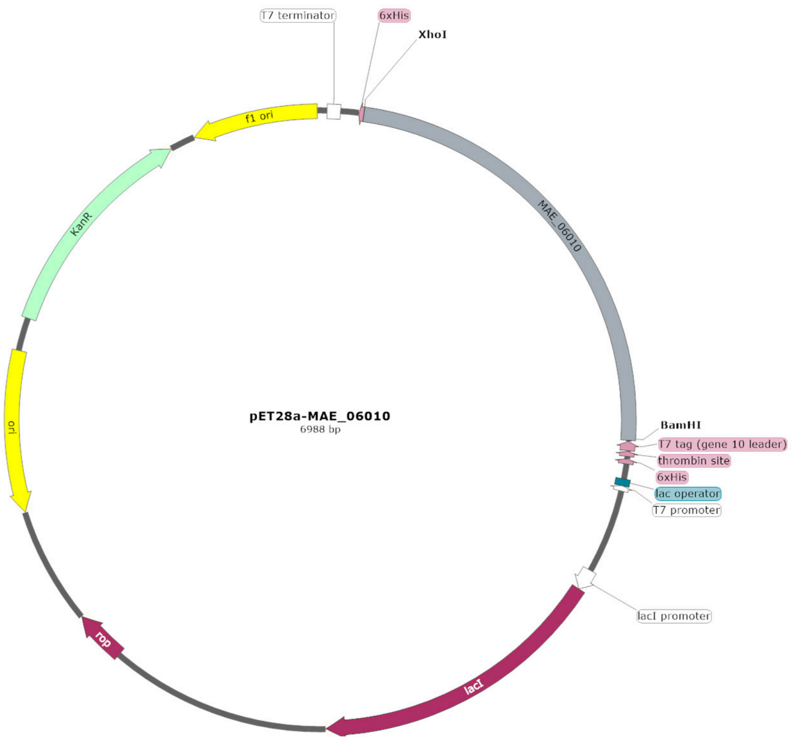

Figure S13. Plasmid map of pET28a-*MAE_06010* plasmid.

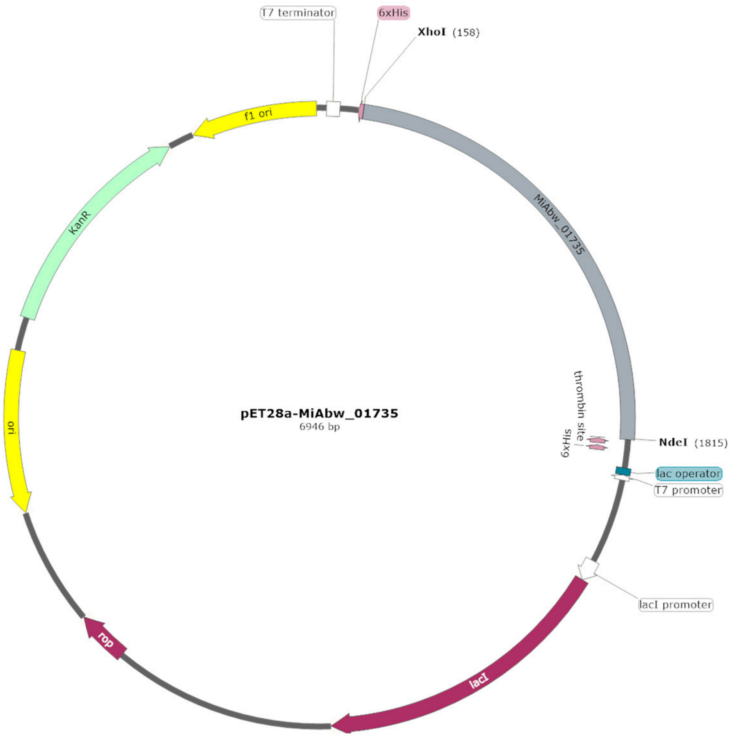

Figure S14. Plasmid map of pET28a-*MiAbW_01735* plasmid.
